# Risk Factors of Very Severe RSV Infections in a Multicenter Cohort of Very Preterm and Extreme Preterm Babies Receiving or Not Palivizumab

**DOI:** 10.3389/fped.2022.884120

**Published:** 2022-07-07

**Authors:** Gwenaelle Mulot, Mehdi Benchaib, Frank Plaisant, Dominique Ploin, Yves Gillet, Etienne Javouhey, Olivier Claris, Jean-Charles Picaud, Jean-Sebastien Casalegno, Marine Butin

**Affiliations:** ^1^Hospices Civils de Lyon, Hôpital Femme Mère Enfant, Service de Réanimation Néonatale, Bron, France; ^2^Hospices Civils de Lyon, Hôpital Femme Mère Enfant, Service de Médecine de la Reproduction, Bron, France; ^3^UMR CNRS 5558 – LBBE, Villeurbanne, France; ^4^Hospices Civils de Lyon, Hôpital Femme Mère Enfant, Service de Réanimation et Urgences Pédiatriques, Bron, France; ^5^Centre International de Recherche en Infectiologie (CIRI), Team VirPatH, INSERM U1111, CNRS UMR5308, Ecole Normale Supérieure de Lyon, Université Claude Bernard Lyon 1, Lyon, France; ^6^Centre International de Recherche en Infectiologie, Team Staphylococcal Pathogenesis, INSERM U1111, CNRS UMR5308, Ecole Normale Supérieure de Lyon, Université Claude Bernard Lyon 1, Lyon, France; ^7^EA 4129, Université Claude Bernard Lyon 1, Lyon, France; ^8^Hospices Civils de Lyon, Hôpital de la Croix Rousse, Service de Réanimation Néonatale, Lyon, France; ^9^CarMeN, INSERM U1060, INRA U1397, Université Claude Bernard Lyon 1, Pierre-Bénite, France; ^10^Institut des Agents Infectieux, Hôpital de la Croix Rousse, Hospices Civils de Lyon, Lyon, France

**Keywords:** cohort study, RSV, bronchiolitis, preterm, palivizumab, LRTI

## Abstract

**Introduction:**

Preterm infants are at risk of lower respiratory tract infections (LRTI), including Respiratory Syncytial Virus (RSV) associated bronchiolitis, for which palivizumab prophylaxis can be proposed. Our aim was to determine risk factors of very severe RSV disease in children born before 34 weeks of gestation.

**Methods:**

Among 2,101 infants born before 34 weeks of gestation in 3 maternity wards between 2012 and 2017, the laboratory confirmed RSV-infected patients requiring hospitalization before 12 months of corrected age were retrospectively included. We collected data about the neonatal period, the palivizumab prophylaxis and the hospitalization for a RSV-related LRTI. LRTI was considered as very severe (VS-LRTI) when patients required invasive or non-invasive positive pressure ventilation.

**Results:**

Among 86 included patients, 31 met the criteria of VS-LRTI. The VS-LRTI patients had a higher birth gestational age and weight but less heart disease and bronchopulmonary dysplasia. They received palivizumab prophylaxis less frequently than the other patients but the difference was not significant. At the onset of infection, VS-LRTI patients had a younger corrected age for prematurity and presented more frequently with apnea, bradycardia, life-threatening event, hemodynamic failure, hypercapnia. Using logistic regression, the main factor associated with VS-LRTI was a younger corrected age for prematurity at the onset of infection [Odd ratio for each month of corrected age = 0.77 (0.62; 0.93), *p* = 0.012].

**Conclusion:**

Infants at the highest risk of VS-LRTI were infants with a younger corrected age for prematurity. Therefore, a better targeting of infants requiring palivizumab prophylaxis and early interventions at hospital discharge could limit VS-LRTI in these infants.

## Introduction

Despite an outstanding improvement in the neonatal intensive care, preterm birth complications remain the leading cause of death under 5-year-old (1). Preterm infants face multiple complications at short, mid or long term and are at risk to develop bronchopulmonary dysplasia, causing an alteration of the constitution of the lung and of its function (2). Given this pulmonary vulnerability, preterm infants are at risk to develop lower respiratory tract infections (LRTI) including Respiratory Syncytial Virus (RSV) associated bronchiolitis.

RSV is responsible for a high burden in young infants during winter outbreaks each year. It represents the most common cause of hospital admission for acute LRTI and in France 30% of children under 2 years old are infected each year, of which 2% require hospitalization (3). Among RSV-infected patients, preterm infants are at higher risk of complications, hospitalization in Pediatric Intensive Care Unit (PICU) and non-invasive and invasive ventilation (4). Each year 63.8 hospitalizations and 1.04 deaths per 1,000 premature children are related to an RSV infection worldwide (5). To reduce the prevalence and burden of this disease, palivizumab, a humanized monoclonal antibody against the RSV F protein is recommended in population at risk of severe outcome, including very preterm infants and infants with bronchopulmonary dysplasia or hemodynamically significant congenital heart disease (6).

The subtle identification of the factors associated with the occurrence of severe RSV infection could enhance the prevention and management of such disease, including palivizumab but also targeted prevention. In the overall population, risks of severe disease have been explored and are prematurity, a younger age, the presence of a comorbidity, environmental factors and viral co-infection (7). Moreover, some studies have focused on infants born at 33–35 weeks of gestation to discuss the interest to extend indications of palivizumab in high-risk late preterm babies (8). However, if such factors are well identified in term and late-preterm neonates, few data are available concerning the risk factors of severe RSV infections in very and extreme preterm babies.

The aim of the present study was to determine risk factors of very severe RSV disease in a population of infants born before 34 weeks of gestation who were hospitalized for an RSV infection.

## Methods

A large database including all neonates born in one of the 3 maternity wards of the University Hospital of Lyon (HCL; Hospices Civils de Lyon) from January 01, 2012 to December 31, 2017, with parents living in Lyon or the suburb areas was built as previously described (9). In Lyon and its suburb areas, the *Hôpital Femme Mère Enfant* (HCL) is the single pediatric hospital with a PICU ward, so it centralizes all infants requiring hospitalization at risk of organ failure, especially all ex preterm babies. The quasi-exhaustivity of the birth cohort allows considering the cohort and the management of hospitalized patients presented herein as representative of the whole cohort.

For the purpose of the present study, we focused on infants born before 34 weeks of gestation. This cohort was cross-referenced with the database of the HCL laboratory to identify ex preterm infants who were subsequently hospitalized in the *Hôpital Femme Mère Enfant* for an RSV infection. Of note, in this hospital all infants with a LRTI requiring hospitalization are swabbed for viral test and RSV is systematically searched by RT-PCR. Infants who were hospitalized before 12 months of corrected age for prematurity were included in this retrospective cohort study. Corrected age is the age calculated by taking the chronological age of the infant and subtracting the number of weeks the infant was born prior to term.

Data concerning the perinatal and neonatal periods were collected from the ICCA software (Philips^®^, Suresne, France) which prospectively encodes medical information during NICU hospitalization until home discharge. Collected data included birth conditions and parameters, complications (cardiac, respiratory, neurological), presence of siblings, month and age at discharge, administration of palivizumab prophylaxis. Bronchopulmonary dysplasia was defined as oxygen and/or positive pressure ventilation requirement at 36 weeks (2). Small for gestational age was defined as a birth weight <10th percentile for gestational age. Data concerning the RSV infection were retrieved by reviewing the medical records of the emergency room visit and hospitalization. Data about the environment (precariousness, passive smoking, breastfeeding at the onset of bronchiolitis, childcare in the community) were collected. Breastfeeding was considered even if not exclusive. Precariousness was defined by numerous situations that were identified in the medical records, including migrant family, homeless family, single-parent family, unemployed parents, etc. The clinical presentation at the LRTI onset, the presence of viral co-infections, the laboratory and X-ray findings, the management including the ward of hospitalization (PICU or pediatric ward), the need for ventilation and/or oxygen supply, the administration of antibiotics, bronchodilators or corticosteroids were also collected.

LRTI was considered as very severe (VS-LRTI) when patients required a positive pressure ventilation (including mechanical ventilation, CPAP, NIPPV, high flow nasal cannula) with or without oxygen. LRTI requiring only passive oxygen using hood/tent or low flow cannula (without positive pressure), were not considered as very severe.

Statistical analysis was performed using R version (Cran, v3.8). Quantitative variables were expressed as medians and interquartile range (IQR) or mean ± standard deviation, qualitative variables as numbers and percentages. VS-LRTI and non VS-LRTI groups were compared using *t*-test (quantitative variables with equal variances), welch test (quantitative variables with unequal variance), Mann-Whitney test (quantitative variables with a not normal distribution) or chi two test (qualitative variables).

To identify risk factors of VS-LRTI among ex preterm patients hospitalized for a RVS-related infection, a logistic regression was performed, with an adjustment for the variables with a significant difference between the 2 groups of patients and for clinically relevant variables. Of note the gestational age, the birth weight and the postnatal age were not included since the item “corrected age” was directly linked to these items and was included in the regression model. The administration of palivizumab was included in the model given the previous literature data showing its impact in decreasing the VS-LRTI (8). The factor “childcare in the community” was not included since it was directly linked to the corrected age: in France the maternity leave begins at birth and ends at 10 weeks after the theoretical date of term, and children are not in the community during this period so the younger patients were never exposed to this confounding factor.

A *p*-value under 0.05 was considered as significant.

The data file was registered to the French data protection authority (*Commission Nationale de l'Informatique et des Libertés*, MR003 N°18-07304/05/2018). This study was observational and used anonymous data. In accordance with French regulations, parents were informed of the study by postal mail, and were given the possibility to refuse to participate, but Ethics Committee approval was not required.

## Results

**Figure 1:**
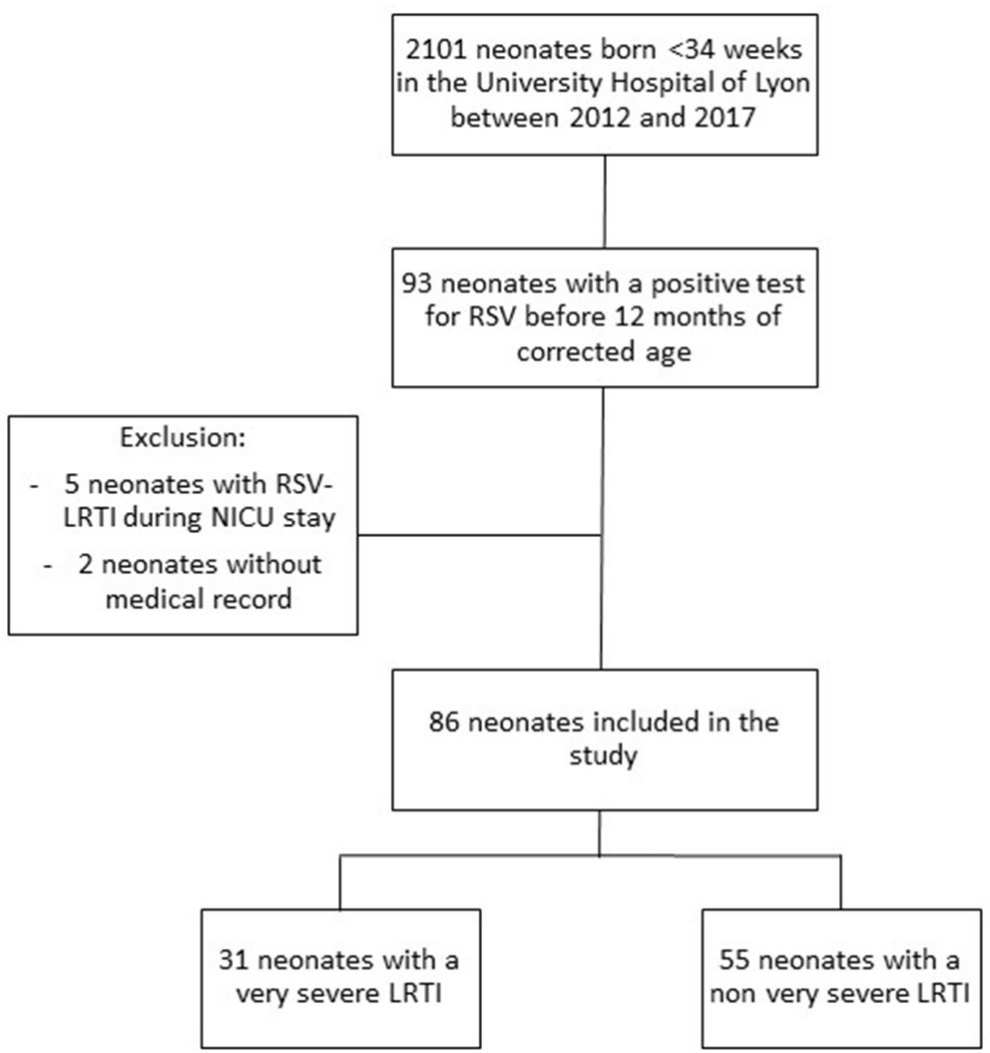
Flow chart of the study cohort, including all ex preterm infants born before 34 weeks of gestation in one of the 3 maternity wards of the University Hospital of Lyon between 2012 and 2017.

During the 6-year study period, 2,101 neonates were born before 34 weeks of gestation in the University Hospital of Lyon. Mean gestational age was 30.2 weeks and mean birth weight was 1,460 grams. Sixteen percent (*n* = 325) of the patients presented with a bronchopulmonary dysplasia. Before 12 months of corrected age for prematurity, an hospitalization related to RSV-LRTI occurred in 93 patients. Five patients were excluded from the analysis because this infection occurred during the NICU hospitalization and two because the medical record was not retrieved. No parents refused participation. Thus, 86 patients were included in the present analysis, 31 of them meeting the criteria of VS-LRTI (Figure 1).

At birth, patients in the VS-LRTI group had a higher birth gestational age and birth weight but less congenital heart disease and bronchopulmonary dysplasia (Table 1). A lower proportion of patients in the VS-LRTI group received palivizumab but the difference between the 2 groups was not statistically significant (*p* = 0.051).

**Table 1:** Medical history and demographic characteristics of the 86 preterm babies hospitalized for a RSV-LRTI in Lyon between 2012 and 2017.

	**Very severe**	**Non very severe**	***p***
	**RSV-LRTI**	**RSV-LRTI**	
	**(*n* = 31)**	**(*n* = 55)**	
Gestational age (weeks)	30.8 (±1.75)	29.5 (±2.67)	<0.01
Birth weight (grams)	1,602 (±472)	1,333 (±470)	0.014
Small for gestational age	3 (9.7%)	10 (18%)	0.36
Boys	19 (61%)	27 (49%)	0.28
Vaginal delivery	14 (45%)	13 (24%)	0.039
Bronchopulmonary dysplasia	3 (10%)	16 (30%)	0.035
Congenital heart disease	0 (0%)	9 (17%)	0.023
Precariousness	9 (29%)	9 (16%)	0.17
Passive smoking	8 (33%)	22 (46%)	0.31
Breastfeeding	7 (23%)	11 (20%)	0.78
Community childcare	1 (3.4%)	12 (28%)	<0.01
Siblings	23 (74%)	36 (72%)	0.83
Palivizumab prophylaxis	7 (23%)	24 (44%)	0.051

*Date are expressed as mean (±SD, standard deviation) and n (%)*.

At the onset of infection, patients with VS-LRTI were younger (corrected age for prematurity 1.52 vs. 4.02 months in the non VS LRTI group, *p* < 0.01) (Table 2). Apnea, bradycardia, life-threatening event, hemodynamic failure were more frequent in the VS LRTI patients, as well as hypercapnia and high C-reactive protein (CRP) value.

**Table 2:** Baseline characteristics at the onset of hospitalization in 86 preterm babies hospitalized for a RSV-LRTI in Lyon between 2012 and 2017.

		**Very severe**	**Non very severe**	***p***
		**RSV-LRTI**	**RSV-LRTI**	
		**(*n* = 31)**	**(*n* = 55)**	
Corrected age for prematurity (months)		1.52 (±2.63)	4.02 (±3.54)	<0.001
Chronological age (months)		3.26 (±2.78)	6.20 (±3.88)	<0.001
RSV season of infection	2012	6 (19%)	6 (11%)	0.048
	2013	4 (13%)	12 (22%)	
	2014	1 (3.2%)	8 (15%)	
	2015	12 (39%)	9 (16%)	
	2016	1 (3.2%)	9 (16%)	
	2017	7 (23%)	11 (20%)	
Apnea and/or cyanosis		16 (52%)	10 (18%)	<0.01
Bradycardia and/or life-threatening event		14 (45%)	7 (13%)	<0.001
Respiratory distress		29 (97%)	48 (87%)	0.25
Cough		21 (68%)	40 (73%)	0.62
Hemodynamic failure		10 (32%)	7 (13%)	0.029
Reduced food intake		28 (90%)	42 (76%)	0.11
Fever		10 (32%)	27 (49%)	0.13
Hypercapnia > 6 kPA		27 (93%)	4 (24%)	<0.001
Hyperleukocytosis > 17 G/L		5 (23%)	5 (28%)	0.73
Hyponatremia <135 mmol/L		5 (20%)	0 (0%)	0.056
CRP highest value (mg/L)		49.5 (12.3; 105.0)	17.0 (2.0; 44.6)	0.049
Acidosis (pH <7.35)		24 (83%)	3 (18%)	<0.001

*CRP, C-reactive protein*.

*Date are expressed as mean (±SD, standard deviation), n (%) or median (interquartile range)*.

All patients requiring ventilation support were in the VS-LRTI group according to the definition of this group (Table 3). Among the 31 patients with a VS-LRTI, 6 (19%) required mechanical ventilation. Corticosteroids and bronchodilators were more frequently administered in non VS-LRTI patients than in VS-LRTI patients.

**Table 3:** Management of the 86 preterm babies hospitalized for a RSV-LRTI in Lyon between 2012 and 2017.

	**Very severe**	**Non very severe**	***p***
	**RSV-LRTI**	**RSV-LRTI**	
	**(*n* = 31)**	**(*n* = 55)**	
PICU admission	29 (94%)	5 (9.1%)	<0.001
Oxygen hood/tent	10 (32%)	8 (15%)	0.053
Low flow oxygen	20 (65%)	28 (51%)	0.22
High flow nasal canula	20 (65%)	0 (0%)	<0.001
CPAP	19 (61%)	0 (0%)	<0.001
NIPPV	15 (48%)	0 (0%)	<0.001
Non-invasive NAVA	7 (23%)	0 (0%)	<0.001
Mechanical ventilation	6 (19%)	0 (0%)	<0.01
Highest level of FIO2 (fraction)	0.30 (0.28; 0.51)	NA	NA
Salbutamol administration	9 (29%)	35 (64%)	<0.01
Antibiotics administration	17 (55%)	20 (36%)	0.097
Corticosteroids administration	8 (26%)	33 (60%)	<0.01
Length of stay (days)	12.0 (6.5; 19.5)	4.0 (2.0; 6.8)	<0.001

*PICU, pediatric intensive care unit; CPAP, continuous positive airway pressure; NIPPV, Nasal intermittent positive pressure ventilation; NAVA, Neurally adjusted ventilatory assist; FIO2, fractional concentration of inspired oxygen; NA, not available*.

*Highest level of FiO2 was available only for patients of the very severe RSV-LRTI group*.

*Date are expressed as n (%) or median (interquartile range)*.

In the logistic regression analysis, the main factor associated with a VS-LRTI in ex preterm patients was a younger corrected age at the onset of infection [Odd ratio for each month of corrected age = 0.77 (0.62; 0.93), *p* = 0.012] (Table 4).

**Table 4:** Logistic regression model exploring the risk factors of very severe LRTI in the cohort of 86 preterm babies hospitalized for a RSV-LRTI in Lyon between 2012 and 2017.

		**Odds-ratio (95% CI)**	***p***
Corrected age for prematurity (months)		0.77 (0.62; 0.93)	0.012
Bronchopulmonary dysplasia	No	Reference	–
	Yes	0.56 (0.09; 3.05)	0.510
Palivizumab	No	Reference	–
	Yes	0.74 (0.19; 2.68)	0.650

*95% CI, 95% confidence interval*.

## Discussion

In the present study, we explored the risk factors of VS-LRTI in a cohort of 86 ex preterm patients hospitalized for RSV infection during their first year of corrected age. Based on a logistic regression model we showed that a younger corrected age for prematurity at the onset of infection was the main factor associated with VS-LRTI.

The impact of a young corrected age on the severity of the LRTI is consistent with previous findings (10). This can be explained by the pathophysiology of bronchiolitis: epithelial injuries related to the virus can lead to mucus accumulation and bronchiole obstruction that is increased in the smallest babies who present with a low diameter of the bronchioles. Moreover, the viral load in the respiratory tract is increased in the younger infants because of a low production of specific antibodies (11). The implementation of advices and prophylaxis should target the neonates directly at the hospital discharge since the younger age is the main risk factor of very severe infection. Surprisingly, concerning strategies of prevention, if literature is abundant about monoclonal antibodies or possibilities of maternal-fetal immunization using vaccines during pregnancy (12), data are lacking about simple methods of prevention including masks, hydroalcoholic gel and education of parents. We could extrapolate strategies from the recent experience of COVID19 pandemics. In this context, a study highlighted that application of preventive measures was higher if the patient had a high risk perception, and authors concluded that developing effective health education programs and frequent communication strategies are necessary (13). Similar preventive strategies should be recommended for RSV prevention, since it has been shown a significant decrease of RSV prevalence during COVID19 pandemics, suggesting that these preventive measures are also active against RSV transmission (14).

In our population-based study of infants born before 34 weeks of gestation, we observed a hospitalization rate of 4.4%. It is less than the data of a previous meta-analysis (5) who reported a global incidence of RSV associated hospitalization among preterm infants of 6.38 per 100 children, 3 times greater than for term children <1 year of age. Moreover, in a cohort of 2,571 French children born before 32 weeks of gestation, Charkaluk et al. found an incidence rate for LRTI of 52.2% at 1-year corrected of age, but the cases were identified by parental questionnaire and included both hospitalized and non-hospitalized episodes (15). The slight difference of hospitalization rate could be the consequence of focusing the analysis on only one hospital in our study. In addition, our study focused on RSV-related bronchiolitis, whereas other studies included all bronchiolitis.

The lower incidence of congenital heart disease, bronchopulmonary dysplasia or low gestational age at birth in the VS LRTI group was an unexpected result. However, this could be a direct effect of the palivizumab prophylaxis, which targets especially these infants at risk of RSV VS-LRTI. This hypothesis is reinforced by the fact that in our study the incidence of palivizumab prophylaxis was lower in the VS LRTI group. We can assume that children at higher risk received the prophylaxis, which prevented them from hospitalization, and therefore are not part of the VS LRTI group. Those results are consistent with those of Butt et al. who found that more than 88% of PICU admission with RSV infection would not qualify for RSV prophylaxis, although the study had been conducted among premature and term infants (16). The recommendations for palivizumab prophylaxis in the NICUs of our region are either a gestational age at birth <29 weeks or a gestational age at birth between 29 and 32 weeks with bronchopulmonary dysplasia and another risk factor (siblings, growth restriction, passive smoking) or congenital abnormalities at high risk of LRTI (hemodynamically significant congenital heart disease, diaphragmatic hernia, etc.) (17). Among the 24 patients of our cohort with a VS LRTI who did not receive palivizumab prophylaxis, only 2 fulfilled these indications of palivizumab (one born at 28 gestational weeks and one born at 30 gestational weeks with bronchopulmonary dysplasia and siblings, to whom prophylaxis have not been proposed).

In a previous study Charkaluk et al. found community care and the presence of siblings to be risk factors of LRTI before 1-year corrected age among their cohort of very preterm infants (15). This was not observed in our cohort, but this result could be correlated with the mean corrected age of 1.5 months at hospitalization in the VS LRTI group. A very few proportions of children are in community in this group since maternity leave's duration in France is 10 weeks after pregnancy term, so the younger infants are usually cared at home.

Interestingly, the study of the clinical characteristics of children in the VS LRTI group vs. the non VS LRTI group distinguishes two profiles. The VS LRTI patients mainly presented with apnea, bradycardia, life-threatening event and hemodynamic failure, whereas non VS LRTI were older, with a frequent cough and probably wheezing presentation since they frequently received corticosteroid and bronchodilatators. Previously, Ghazaly et al. showed that 66% of premature children hospitalized in the PICU for bronchiolitis presented with apnea (18). Moreover, another study concluded that age below 2 months was the strongest independent risk factor for RSV associated apnea (19). It can be explained by the immaturity of the respiratory center of the brain, notably among very premature children and the presence of larynx's chemoreceptors sensitive to RSV.

Of note, PICU admission was not necessarily a marker of severity since in the study center the decision of PICU admission was based on either the clinical severity of the patients, or the vulnerability of the patient. Thus, some patients of the non-very severe RSV-LRTI group were admitted to the PICU, due to their vulnerability including for example extremely preterm birth, even if their clinical status was stable.

The last point that we have observed is the differences of severity between the 6 seasons of RSV outbreaks. This has already been reported and seems to be related to the main viral strain that is circulating each season (20).

This study has several limitations. First, the retrospective design of the study could lead to missing data. This is particularly true for some data that were not systematically recorded (breastfeeding, type of childcare, precariousness), limiting the possibility to analyze those items. Secondly, the decision of ventilation or oxygen therapy (which was our primary outcome) is clinical and therefore submitted to personal judgment of the physician in charge, which in the context of high prematurity can be increased. Third, we focused on RSV infection but it could have been interesting to include all LRTI whatever was the virus involved. Finally, it is possible that we have lost of follow up some neonates that were born in one of the maternity wards of the University Hospital of Lyon, but hospitalized in another hospital outside Lyon area.

## Conclusion

Efforts to better understand the risk factors associated with severity in RSV related LRTI in very premature children are necessary to elaborate more adapted preventive interventions based on early interventions strategies and monitoring. A particular consideration should be given to the youngest infants who are the population at the highest risk of severe infection. Palivizumab is an expensive prophylactic strategy so it cannot be generalized to the whole population of neonates. Moreover, it is not sufficient to avoid RSV infection. Educational approach of the families at the hospital discharge could help in decreasing the rate of RSV transmission to vulnerable young infants. To improve compliance to the preventive measures, a “RSV prevention kit” could also be given to parents with documentation, masks and hydroalcoholic gel, at the hospital discharge.

## Data Availability Statement

The raw data supporting the conclusions of this article will be made available by the authors, without undue reservation.

## Ethics Statement

Ethical review and approval was not required for the study on human participants in accordance with the local legislation and institutional requirements. Written informed consent from the participants' legal guardian/next of kin was not required to participate in this study in accordance with the national legislation and the institutional requirements.

## Author Contributions

GM and FP: acquisition of the data. GM, MBe, MBu, and J-SC: interpretation of the data. GM and MBu: draft of the article. All authors: revision of the manuscript and final approval of the article.

## Conflict of Interest

The authors declare that the research was conducted in the absence of any commercial or financial relationships that could be construed as a potential conflict of interest.

## Publisher's Note

All claims expressed in this article are solely those of the authors and do not necessarily represent those of their affiliated organizations, or those of the publisher, the editors and the reviewers. Any product that may be evaluated in this article, or claim that may be made by its manufacturer, is not guaranteed or endorsed by the publisher.
